# Editorial: The Use of Organoids in Cancer Biology

**DOI:** 10.3389/fcell.2022.948926

**Published:** 2022-07-07

**Authors:** Xiang Xue, Julie In, Hua Geng, Ying Xiao, Zhangfa Song

**Affiliations:** ^1^ Department of Biochemistry and Molecular Biology, School of Medicine, University of New Mexico, Albuquerque, NM, United States; ^2^ Division of Gastroenterology, Department of Internal Medicine, School of Medicine, University of New Mexico, Albuquerque, NM, United States; ^3^ Ann and Robert H. Lurie Children’s Hospital of Chicago, Department of Pediatrics, Northwestern University, Chicago, IL, United States; ^4^ Department of Colorectal Surgery, Sir Run Run Shaw Hospital, School of Medicine, Zhejiang University, Hangzhou, China

**Keywords:** organoids, cancer, biobank, metastasis, therapy

Cancer is a major public health problem worldwide and the second leading cause of death in the United States (Siegel et al., 2022). Despite the advance in medicine, there is still no therapeutic that can cure advanced cancer. Thus, better therapeutic approaches are urgently needed for targeting cancer cells. Organoids are an emerging research tool in stem cell biology, regenerative medicine, virology, and cancer biology. This editorial article frames the aim of this Research Topic as to gather promising, recent, and novel research trends in the organoids towards cancer biology field.

There are two approaches to establish organoids. The first approach was developed by the Hans Clevers’ group. They established the long-term culture conditions for self-organizing crypt-villus structures named organoids that proliferate *in vitro* without a mesenchymal niche (Sato et al., 2009). These organoids are generated from single sorted Lgr5 (+) stem cells isolated from crypts obtained from adult donors (Sato et al., 2009). The second approach was developed by the James Wells’ group. They successfully differentiated human embryonic and induced pluripotent stem cells (iPSCs) into three-dimensional intestinal ‘organoids’ *in vitro* (Spence et al., 2011). These iPSC organoids are also surrounded by mesenchymal cells that secrete factors found in the mesenchymal niche. Both approaches are used by researchers, but the first approach is more easily applicable for tissues from various organs, takes a shorter time to establish and the culture media has been commercialized. One of the research articles published in this Research Topic alerts us that low-level contaminating mouse DNA is present in the conditioned culture media derived from the L-WRN cell line and in human organoid cultures maintained in this media (Bohm et al., 2020). This suggests that we still need to optimize the culture conditions. Moreover, as discussed in one of the review articles published in this Research Topic (Luo et al., 2022), organoid biobanks are established at both academic institutes (Bender, 2015), and nonprofit organizations such as American Type Culture Collection (ATCC).

Organoids are highly physiologically relevant models and appear as an ideal system to recapitulate many of the central aspects of tumor microenvironment including, but not limited to, 3D morphology and polarized expression of differentiation and stem cell markers. Many such markers are lost in established cell lines. Furthermore, tumor organoids can be genetically engineered using virus-mediated gene delivery or generated from genetically modified animals. Importantly, a subset of the patient-derived organoids can be grown in minimally supplemented serum-free media, which is desirable for experimental interrogations such as nutrient supplementation. Recent developed the long-term homeostatic culture of tubular mini-guts which overcomes the limitation of traditional 3D organoids developing tissues with a closed, cystic architecture that restricts lifespan and size, makes the organoids can be experimental manipulated and prolong its homeostasis (Nikolaev et al., 2020).

Human colon organoids are useful for both physiologic and pathophysiologic studies, because they are a tractable intestinal epithelia model that better mimic the gut compared to cell lines. Importantly, it has been observed that epithelial-produced WNT2B is essential for colonic regeneration and works with the Hedgehog pathway in human organoids (In et al., 2020). This partly explains the contribution of Wnt and Hedgehog signaling in injury-induced colonic regeneration and in the initiation steps of left-sided colorectal cancer. Moreover, iron is required for sustaining nucleotide metabolism and mitochondrial function in patient-derived colorectal cancer organoids (Schwartz et al., 2021). Consistently, pharmacological inhibition of iron uptake transporter divalent metal transporter 1 (DMT1) reduces tumor growth and cell proliferation of patient-derived colorectal cancer organoids *in vitro* and in an orthotopic transplantation mouse model (Xue et al., 2016).

Metastasis is the primary cause of morbidity and mortality in cancer patients. Investigators have developed sophisticated genetic engineered mouse models to recapture tumor progression and metastasis (Tauriello et al., 2018). However, the breeding protocol is arduous and is not cost-effective. Alternatively, tumor organoids derived from these mouse models have been established to study metastasis within a few weeks. Orthotopic transplantation models of CRISPR-Cas9-edited mouse organoids and patient-derived organoids have also been established with efficient and rapid metastatic tumor formation (Roper et al., 2017).

Patient-derived tumor organoids can faithfully recapitulate many characteristics of *in vivo* tumors. This has enabled the determination of individualized drug responses through organoid screening (Toshimitsu et al., 2022). One of the research articles published in this Research Topic investigated the effects of a multi-mineral natural product on colonic barrier in colonoids and suggested that adequate mineral intake might improve the colonic barrier (Aslam et al., 2020). More and more researchers are also using 3D-organoids-derived monolayer culture for drug screening and other experiments (Kozuka et al., 2017). Furthermore, the baseline properties of tumor organoids predict immunological response to PD-1 inhibition (Voabil et al., 2021), thus facilitating immunotherapy.

In Summary, organoids are a promising and powerful research tool in oncology research. As discussed in one of the review articles published in this Research Topic (Yang et al., 2021), there are still challenges and limitations in organoids application, but with the advanced bioengineering techniques such as 3D Bioprinting and Organoid-on-a-Chip, we expect a common use of organoids in clinical application will be realized in the near future.
